# Supplementary material for: Are Healthcare Workers Infected with SARS-CoV-2 at Home or at Work? A Comparative Prevalence Study
Source: Int J Environ Res Public Health. 2022 Oct 10;19(19):12951. doi: 10.3390/ijerph191912951 (PMC9564591; doi:10.3390/ijerph191912951)
Supplement: Supplementary file 1 [file ijerph-19-12951-s001.zip › ijerph-1940627-supplementary.pdf]

**Table S1:** Relative Rate Ratios for Hadassah Workers by Neighbourhoods of residence.						
**Neighbourhood**	Adult Population	Positive Adult	Hadassah Workers	Hadassah Positive Cases	RR (CI)	Subgroup
**Neighbourhoods with More than 200 workers**						
**H01**	16561	477	397	24	2.1 (1.4 - 3.1)	Jewish
**H02**	31976	915	371	22	2.1 (1.4 - 3.1)	Jewish
**H03**	23824	630	237	4	0.7 (0.3 - 1.7)	Jewish
**H04**	10350	171	217	14	4.0 (2.4 - 6.7)	Jewish
**Neighbourhoods with 100-200 workers**						
**H05**	14313	196	198	4	1.6 (0.7 - 4.1)	Jewish
**H06**	16615	421	191	14	3 (1.8 - 4.9)	Jewish
**H07**	29076	1196	153	20	3.2 (2.2 - 4.8)	Arab
**H08**	11053	390	136	11	2.4 (1.4 - 4.1)	Jewish
**H09**	3482	69	135	13	5 (2.9 - 8.7)	Jewish
**H10**	9747	421	111	15	3.2 (2.0 - 5.1)	Arab
**H11**	15542	669	109	17	3.7 (2.4 - 5.7)	Arab
**H12**	7589	112	106	6	4.1 (1.9 - 8.8)	Jewish
**H13**	8302	121	102	4	3.0 (1.2 - 7.5)	Jewish
**H14**	30664	2077	102	4	0.6 (0.3 - 1.6)	Orthodox Jewish
**Neighbourhoods with 50-100 workers**						
**H15**	3327	77	96	1	0.7 (0.1 - 3.3)	Jewish
**H16***	45	0	93	6	6.3 (0.4 – 10.9)	Jewish
**H17**	12106	207	90	4	2.9 (1.2 - 7.2)	Jewish
**H18**	13687	554	90	9	2.6 (1.4 - 4.7)	Arab
**H19**	24053	874	73	13	5.0 (3.1 - 8.2)	Arab
**H20**	1623	16	70	4	6.3 (2.3 - 17.0)	Jewish
**H21**	20076	969	64	9	3.0 (1.7 - 5.5)	Arab
**H22**	5311	96	64	3	3 (1.1 - 8.4)	Jewish
**H23**	2917	32	64	0	0.7 (0.1 - 11.0)	Jewish
**H24**	6605	104	64	4	4.4 (1.8 - 10.0)	Jewish
**H25**	8834	141	63	2	2.5 (0.7 - 8.3)	Jewish
**H26**	5960	216	61	2	1.1 (0.3 - 3.8)	Jewish
**H27**	14751	419	60	6	3.8 (1.8 - 7.8)	Arab
**H28**	11130	569	58	13	4.5 (2.8 - 7.2)	Arab
**H29**	3404	72	56	2	2.1 (0.6 - 7.1)	Jewish
**H30**	14866	1064	54	6	1.7 (0.8 - 3.4)	Orthodox Jewish
**H31**	4885	149	53	1	0.9 (0.2 - 4.4)	Jewish
**Neighbourhoods with less than 50 workers**						
**H32**	7005	146	49	2	2.4 (0.7 - 8.1)	Jewish
**H33**	12336	364	47	6	4.6 (2.2 - 9.5)	Arab
**H34**	9797	388	46	7	4.1 (2.1 - 7.8)	Arab
**H35**	1757	52	43	0	0.4 (0.1 - 6.1)	Jewish
**H36**	3673	127	41	4	3.1 (1.3 - 7.6)	Jewish
**H37**	992	10	41	3	8 (2.5 - 25.0)	Jewish
**H38**	2680	35	37	1	3.0 (0.6 - 14.0)	Jewish

**H39**	12139	510	35	5	3.7 (1.7 - 7.9)	Arab
**H40**	12797	759	33	3	1.8 (0.6 - 4.7)	Orthodox Jewish
**H41**	7476	307	32	5	4.1 (1.9 - 8.8)	Arab
**H42**	1234	33	32	0	0.6 (0.1 - 9.0)	Jewish
**H43**	7259	146	30	3	5.7 (2.1 - 15.0)	Jewish
**H44**	12084	774	27	1	0.8 (0.2 - 4.0)	Orthodox Jewish
**H45**	2717	38	26	2	6.7 (2 - 22.0)	Jewish
**H46**	7750	564	22	3	2.1 (0.8 - 5.6)	Orthodox Jewish
**H47**	18721	737	20	4	5.6 (2.5 - 12.0)	Arab
**H48**	1663	10	20	0	3.9 (0.2 - 63.0)	Jewish
**H49**	1739	52	18	1	2.7 (0.6 - 12.0)	Jewish
**H50**	1856	48	17	0	1.1 (0.1 - 17.0)	Jewish
**H51**	4778	187	16	2	3.9 (1.2 - 12.0)	Arab
**H52**	9855	822	14	0	0.4 (0.1 - 6.3)	Orthodox Jewish
**H53**	19224	1512	13	1	1.4 (0.3 - 6.3)	Orthodox Jewish
**H54**	3011	54	13	1	6.1 (1.3 - 28.0)	Jewish
**H55**	8144	647	12	2	2.5 (0.8 - 7.6)	Orthodox Jewish
**H56**	2618	168	7	0	1.0 (0.1 - 15.0)	Orthodox Jewish
**H57**	488	4	7	0	7.2 (0.4 - 122)	Jewish
**H58**	9199	518	5	0	1.6 (0.1 - 22.0)	Orthodox Jewish
**H59**	2309	119	5	0	1.8 (0.1 - 24.0)	Jewish
**H60**	4898	235	4	0	2.3 (0.2 - 31.0)	Orthodox Jewish
**H61**	2810	171	4	0	1.8 (0.1 - 24.0)	Jewish
**H62**	3073	88	4	1	11.6 (3.8 - 43.0)	Jewish
**H63**	1802	113	3	1	6.8 (2.0 - 23.0)	Orthodox Jewish
**H64**	2413	39	2	0	12.2 (1.0 - 148)	Jewish
**H65**	729	17	2	1	25.0 (8.2 - 76.0)	Jewish
**H66**	6863	502	1	0	4.5 (0.5 - 43.0)	Orthodox Jewish
**H67**	5195	362	1	0	4.8 (0.5 - 46.0)	Orthodox Jewish
**H68**	3524	227	1	1	15.5 (13.6 - 17.0)	Orthodox Jewish
**H69**	5422	192	1	0	9.4 (1 - 90.0)	Orthodox Jewish
**H70**	722	28	1	0	8.4 (0.8 - 83.0)	Jewish

*H16 – The Hadassah medical center workers dormitory with no corresponding general population.
